# Perspective: Empowering osteoarthritis drug development through assessment of synovitis by CE-MRI: A new approach in clinical trials

**DOI:** 10.1016/j.ostima.2024.100259

**Published:** 2024-12-20

**Authors:** Francis Berenbaum

**Affiliations:** Sorbonne University, INSERM CRSA, AP-HP Saint-Antoine hospital, Paris, France, Saint-Antoine hospital, Department of Rheumatology, 184 rue du faubourg Saint-Antoine, 75012 Paris, France

**Keywords:** Osteoarthritis, Knee, Synovitis, CE-MRI, Surrogate endpoints, Clinical trials

## Abstract

Knee osteoarthritis (OA) is a global health challenge with substantial unmet therapeutic needs. Current treatments primarily target symptoms without altering the disease's progression. Synovitis, the inflammation of synovial tissue, is a key driver of both pain and structural changes in OA. This Perspective proposes a paradigm shift, positioning synovial health assessment as a cornerstone in the evaluation of new OA therapies. By doing so, it aims to accelerate development of effective disease modifying OA drugs (DMOADs) and improving patient outcomes. It highlights the potential of contrast enhanced-MRI (CE-MRI) to serve as a surrogate marker for synovial health, offering precise visualization of inflammation and its relationship with disease progression and pain.

## □

Nearly 600 million people worldwide are affected by osteoarthritis (OA), which is 7 % of the world's population. Of these, 70 % have knee OA [1]. The impact on quality of life is considerable, making it one of the most disabling chronic diseases according to the Global Burden of Disease 2021(1). OA of the knee or hip that affects walking correlates to an increase in mortality of over 50 %, essentially due to the consequences of the sedentary lifestyle imposed by the disease, such as increased cardiovascular events, cancer and dementia. These remarkable figures must be set against our desperate shortage of effective treatments. Of course, weight loss in the case of obesity and physical exercise have proved effective, but we have to admit that we are often at a loss when faced with the most severe forms of the disease. Symptomatic treatments are either ineffective (acetaminophen), or poorly tolerated or contraindicated in these patients (non-steroidal anti-inflammatory drugs (NSAIDs), opioids), who may be elderly, have metabolic comorbidities, or both. Even total knee arthroplasty (TKA) is not a panacea, since in addition to the rare but serious immediate peri‑operative complications (thrombosis, infection), over 20 % of patients continue to take analgesic drugs one year after surgery, but we have no clear understanding of the mechanisms underlying this chronic post-operative pain [2]. As a result, there is considerable unmet need, which has led the FDA to classify OA as a serious condition in 2018, thereby accelerating development programs.

## Reasonably likely surrogate endpoints

This label of serious disease opens up the hope of having at our disposal in the medium-term drugs designed to slow the course of the disease, i.e., to postpone the time until TKA. To begin with, we need to define the “right time for a TKA”, specifically the criteria related to pain, function and structural damage that determine when “it's the right time for a TKA”. This is important because the actual timing depends on many confounding factors other than medical considerations such as cultural and socio-economic influences, which can hamper the interpretation of clinical trials [3]. But the main challenge remains the length of time required for such a clinical trial, which is around 3–5 years [3]. No pharmaceutical company is prepared to invest in such a long trial, with the requirement of including thousands of patients to achieve statistical significance within such a timeframe. The cost is too high for the risk. This is where the FDA's “severe condition” label comes into its own. Indeed, by definition, the possible acceleration of development linked to this label is based on allowing shorter clinical trials, of one to two years, but on the condition that they are based on a surrogate marker of disease progression and, ideally, of the indication for TKA [4]. That is what's at stake. Before a surrogate endpoint can be accepted in place of a clinical outcome, extensive evidence must accumulate, including evidence from epidemiological studies and clinical trials. Usually, clinical trials are needed to show that the surrogate endpoint can be relied upon to predict, or correlate with, clinical benefit in a context of use. This surrogate can be either validated or deemed reasonably likely. It is unrealistic to expect us to be able to validate such a marker in the short term. To do so, we would need a comprehensive database that surpasses the scope of our existing registries, cohorts, and clinical trials, all of which have unfortunately given negative results so far. Indeed, qualification requires not only showing the existence of a correlation between a marker (or a composite of markers) and disease progression, but also demonstrating that an active treatment capable of delaying progression has also modified the marker. The absence of positive randomized trials in this field therefore precludes any validation. On the other hand, finding a reasonably likely surrogate endpoint is the goal [5]. Reasonably likely surrogate endpoints are supported by strong mechanistic and/or epidemiologic rationale, but the amount of clinical data available is not sufficient to show that they are a validated surrogate endpoint. Reasonably likely surrogate endpoints can, however, be used to support FDA's Accelerated Approval program, which is intended to provide faster access to promising therapies to patients with serious diseases [6]. Imaging is certainly one of the most interesting avenues currently open to help us build this reasonably likely surrogate marker, given the vast amount of data now available to us. In particular, data from the two largest American cohorts, Osteoarthritis Initiative (OAI) and Multicenter Osteoarthritis Study (MOST), in addition to extraordinary advances in the pathophysiology of the disease, should help us to define the parameters for a future reasonably likely surrogate marker. With this in mind, on the one hand it seems difficult to envisage a reasonably likely surrogate marker that does not take pain (or a variable strongly correlated with pain) into account, and on the other hand, since OA is now seen as a disease involving all joint tissues (and not just cartilage), we can assume that any drugs acting on one of the joint structures (and not just cartilage) should have an effect on the natural history of the disease.

## Synovial health and pain progression

The most common reason a patient seeks a surgical solution is pain. This is why several studies have found that certain symptomatic treatments, which have no effect on the natural history of the disease, are capable of delaying TKA. This is the case, for example, with hyaluronic acid injections, which have never demonstrated any effect on the disease itself but only relieve symptoms [7].

Unlike synovitis, cartilage degradation has little effect on pain progression. The study by Bacon et al. [8] used OAI data to explore the correlation between cartilage loss and knee pain over a 36-month period, finding that cartilage loss was associated with a very slight worsening of pain, with a remarkably small effect size. These results therefore call into question the assumption that protection against cartilage loss will relieve pain, at least in the short to medium term. This is scientifically logical, since cartilage is not innervated. In contrast, synovial tissue is highly innervated and capable of transmitting pain signals. Recent insights into OA pain mechanisms, as highlighted in the review by Wood et al. underscore the significant roles of synovial tissue in pain perception, distinguishing them from cartilage [9]. Moreover, in the study by Bacon et al. part of the association between cartilage loss and increased pain was mediated by worsening synovitis [8]. This is also the case for bone marrow lesions where synovitis mediates the association between the lesions and knee pain in the OAI cohort [10].

## Synovial health and structural progression

There is now a consensus among the scientific and medical community that OA is a disease that involves all joint tissues, including of course cartilage, but also synovial tissue, subchondral bone, intra-articular fatty tissue, menisci, periarticular muscles and ligaments. Thus, the natural history of the disease can be influenced by the fate of each of these tissues. Synovial tissue, as a structure, therefore, optimally fulfils the definition of a reasonably likely surrogate marker, since it alone is involved both in the transmission of the pain signal and closely correlated with disease progression [11]. Synovitis is related to symptoms, and fluctuations in synovitis correlate with changes in symptoms [12,13]. Synovitis correlates with disease progression and TKA [14]. Synovitis has been identified as a key factor in structural changes early in the disease and is considered an important risk factor for knee OA, as well as for structural progression once the disease is established [[15], [16], [17], [18]]. Synovitis has also been identified as a precursor of cartilage loss in early and established stages of the disease [19].

## Synovial health assessment

MRI allows visualization of soft tissues and fluids in a way that standard radiography does not. As for power Doppler ultrasound, its sensitivity and specificity are inferior for detecting synovitis, and it has limitations in visualizing the detailed and complex structures of the synovial tissue. However, while conventional MRI does provide valuable information, it cannot differentiate intra-articular effusion from the health state of the synovial tissue itself, either in terms of thickness or in terms of inflammatory state. Gadolinium-enhanced MRI (CE-MRI) overcomes these limitations by considerably improving visualization of synovial inflammation, enhancing diagnostic accuracy by delineating the thickness and vascularization of synovial tissue [20]. Synovitis has a strong relation with knee pain severity, and an association between pain and synovitis can be seen much more clearly with CE-MRI than non-CE MRI [12]. In addition, the analysis of synovial thickness and perfusion patterns enabled by CE-MRI provides more precise quantitative information than qualitative or semi-quantitative information [21]. Moreover, gadolinium is well known to be safe [22]. Finally, even if the cost and burden for patients of CE-MRI may seem higher than non-contrast MRI for cartilage assessment, CE-MRI can shorten the time needed for a clinical trial. Limiting the duration of the trial is particularly important for patients randomized into the placebo arm and significantly reduces the economic costs of long, inconclusive clinical trials. Moreover, MRI has the advantage of central readings and less dependence on the experience of the sonographer, for example. It is important, however, to acknowledge that MRI can detect synovial abnormalities even in asymptomatic patients. This may limit its specificity in certain contexts, as noted in the EULAR recommendations that abandoned the use of CE-MRI for diagnosing early inflammatory arthritis due to high rates of positive findings in asymptomatic individuals. Nevertheless, in the context of OA clinical trials—where synovitis is accepted as a key contributor to disease progression—CE-MRI serves as a valuable endpoint to assess therapeutic efficacy, particularly for interventions targeting synovial tissue [23,24]. Finally, other emerging imaging techniques based on CT or MRI, sometimes enhanced by artificial intelligence, have demonstrated their ability to visualize synovitis in various inflammatory joint contexts, with validation awaited in the specific context of OA.

Identifying a reasonably likely surrogate marker is essential for developing disease-modifying treatments that address both symptoms and the natural history of osteoarthritis. This marker will of course need to be customized according to the mechanism of action of the drug being tested, and will probably have to integrate several variables to capture those most likely to predict the hard endpoint within the relatively short timeframe (1–2 years) of a clinical trial. Because recent data from robust, long-term cohorts reinforce that OA progression is not solely driven by cartilage, there is now no doubt that the estimation of synovial tissue health is well placed to be part of these future surrogate markers.
